# The Effects of Flaxseed Consumption on Glycemic Control in Native American Postmenopausal Women with Hyperglycemia and Hyperlipidemia

**DOI:** 10.3390/healthcare12141392

**Published:** 2024-07-11

**Authors:** Ines Ellouze, Kiranmayi Korlagunta, Edralin A. Lucas, Mark Payton, Saiful Singar, Bahram H. Arjmandi

**Affiliations:** 1 Functional Physiology and Bio-Resources Valorization Laboratory, Higher Institute of Biotechnology of Beja, Jendouba University, Beja 9000, Tunisia; 2 Vijayavahini Charitable Foundation, Vijayawada 520008, Andhra Pradesh, India; 3 Nutritional Sciences, College of Education and Human Sciences, Oklahoma State University, Stillwater, OK 74078, USA; 4 Biomedical Sciences, Rocky Vista University, Englewood, CO 80112, USA; mpayton@rvu.edu; 5 Nutrition, Food, and Exercise Sciences, College of Human Sciences, Florida State University, Tallahassee, FL 32306, USA

**Keywords:** flaxseed, postmenopausal, Native American, glucose metabolism

## Abstract

Glucose control in postmenopausal women is influenced by many factors, such as hormones, lifestyle variables, and genetics. Limited data exist on the effect of whole flaxseed on glucose status in postmenopausal Native American women. The aim of this study was to investigate the glucose management effect of a flaxseed dietary intervention on postmenopausal Native American women. In this study, 55 Native American postmenopausal women (aged 47–63 years) with borderline hyperglycemia (>100 and <126 mg/dL) and mild to moderate hypercholestorolemia (≥200 to ≤380 mmol/L), who were not on hormone replacement therapy, were enrolled. Participants were randomly assigned to one of the three dietary regimens (control, flaxseed, and flaxseed + fiber) for three months, receiving interventions in the form of bread, muffins, and flaxseed powder. Despite daily consumption of flaxseed across diverse food formats, no significant changes in glucose (*p* = 0.3, *p* = 0.2), insulin levels (*p* = 0.59, *p* = 0.9), or HOMA-IR (*p* = 0.84, *p* = 0.66) were observed compared to their respective baseline values within the flaxseed and flaxseed + fiber groups, respectively. Conversely, the control group showed a significant rise in final glucose values from baseline (*p* = 0.01). However, the incorporation of ground flaxseed into low-glycemic foods holds potential for beneficial effects through maintaining glucose status among postmenopausal Native American women. This research provides critical insights into the effects of flaxseed, emphasizing the need for continued exploration to understand its role in supporting glucose management among postmenopausal Native American women. Further exploration is required to investigate the potential long-term impact and the use of flaxseed in managing glucose levels in this demographic.

## 1. Introduction

The Centers for Disease Control and Prevention (CDC) estimates that over 38.4 million individuals, with 18.3 million being women, are currently living with diabetes, which represents 11.6% and 5.5% of the US population, respectively [1]. This prevalence is notably higher among minority ethnic groups compared to non-Hispanic Caucasian adults [2]. Within the Native American population, diabetes stands as a significant cause of both mortality and morbidity [3]. Moreover, the repercussions are notably pronounced in certain states, specifically Oklahoma, South Dakota, and North Dakota, affecting approximately 38 to 72% of the Native American population dealing with diabetes [2]. This disease state affects women more severely than men, as various diabetic complications are more likely to occur in women. Indeed, women with diabetes have higher risks of developing cardiovascular diseases as well as hypertension and dyslipidemia. At the same time, they are more likely to develop complications such as kidney disease and depression [4]. Reports [5,6,7,8] suggest that the aging process, particularly associated with menopause, affects lipid metabolism and visceral obesity, both of which contribute indirectly to insulin resistance and type II diabetes development.

Maintaining normal blood glucose levels holds promise for slowing the progression of diabetes-associated macrovascular complications [5,9]. Yet, despite efforts involving diet restriction, modest exercise, and lifestyle changes, it remains challenging to achieve near-normal glycemic profiles in individuals with diabetes [10]. The prolonged use of antidiabetic medications can lead to adverse effects, including hypoglycemia, weight gain, lactic acidosis, and gastrointestinal discomfort [11,12]. Consequently, women in developed countries, such as the US and those in Western Europe, might find substantial benefits in exploring complementary and alternative medicine approaches, particularly functional foods and dietary supplements. These interventions hold promise for preventing and managing chronic diseases, including type II diabetes, cardiovascular conditions, and osteoporosis, among others [13,14].

In terms of whole foods, flaxseed has been shown to have estrogenic [15] to antiestrogenic [16] properties, acting as a weak estrogen agonist when estrogen levels are low (as they can bind to estrogen receptors and mimic the action of endogenous estrogen in the body) and competing for estrogen receptors when levels are elevated (the competitive inhibition can block the stronger natural estrogens from binding), as well as antitumorigenic [17] and antioxidant properties [18]. Notably rich in lignans, α-linolenic acid, and soluble fiber mucilage, flaxseed stands out as a source of significant interest due to its multifaceted health benefits [19], including its impact on the glucose profile of postmenopausal women [20]. It also exhibits potential for combating free radical-associated health problems such as cardiovascular issues, type II diabetes, and inflammatory diseases [21,22,23,24,25,26,27,28,29]. However, there remains a scarcity of studies investigating the specific positive effects of these compounds on carbohydrate metabolism, particularly in Native American postmenopausal women [13]. We hypothesized that the daily incorporation of flaxseed into the diet could potentially exert favorable effects on the glucose regulation of Native American postmenopausal women. This hypothesis was based on two distinct observations: Firstly, the onset of ovarian hormone deficiency during menopause contributes indirectly to alterations in glucose regulation, escalating the susceptibility to type II diabetes among women [5,6,7,8]. Secondly, the lignan compounds inherent in flaxseed bear structural similarities to 17β-estradiol, potentially displaying estrogen-like actions that can positively influence the glycemic profile in postmenopausal women [20]. This randomized controlled trial aimed to examine the impact of whole flaxseed incorporation in daily intake on glucose metabolism in a particular demographic and, therefore, will potentially provide Native American postmenopausal women with a safe and economical option for glucose control.

## 2. Materials and Methods

### 2.1. Study Design

**Figure 1:**
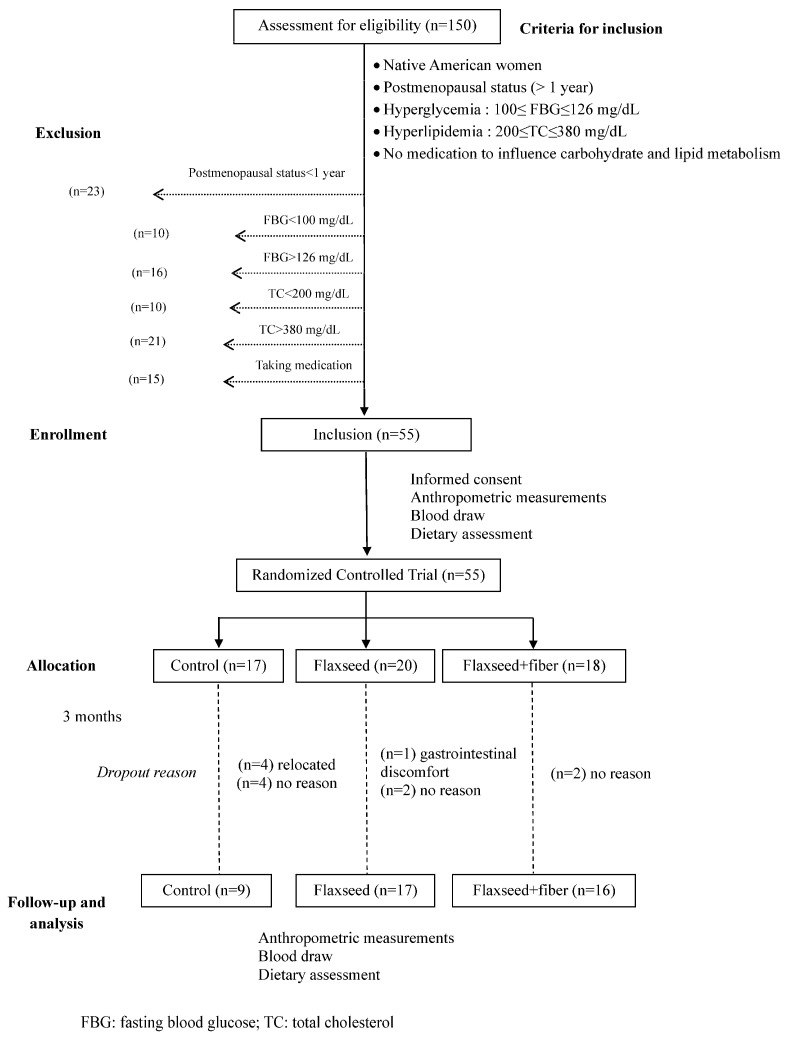
Study design of the randomized controlled trial of flaxseed supplementation.

The experimental design was a randomized controlled trial wherein eligible subjects were randomly assigned into three treatment groups (n = 18 per group): (1) control; (2) flaxseed; and (3) flaxseed + additional fiber for 3 months (Figure 1). The incorporation of the flaxseed + fiber regimen aimed to assess potential synergistic or additive effects on improving glucose profile compared to flaxseed alone, as β-glucan, the main soluble fiber in oat, has an important role in managing glucose and preventing type II diabetes. All the treatment regimens were dispensed biweekly. Participants received guidance on how to adjust their daily food consumption to maintain a consistent caloric intake throughout the study. The detailed composition of the study products is outlined in Table 1. Briefly, the control group consumed 2 oat muffins and 2 slices of bread per day. For the flaxseed and flaxseed + fiber regimens, each subject received 30 g of flaxseed incorporated into bread, muffins, and powder, constituting a part of their daily dietary intake. Participants were instructed to consume 2 muffins (~10 g of flaxseed), 2 slices of bread (~3 g of flaxseed), and 2 tablespoons of flaxseed powder (~16 g) daily. The flaxseed + fiber group received ~8 g extra of soluble dietary fiber from oat bran. The baked products were stored at −20 °C prior to distribution. They were provided to the subjects in a frozen state, and they were instructed to maintain freezing until consumption. Subjects were instructed to thaw these products overnight in the refrigerator or defrost them immediately in a microwave oven before consumption.

### 2.2. Subjects

The study protocol was approved by the Institutional Review Board (IRB) at Oklahoma State University. The inclusion criteria were postmenopausal status confirmed by at least one year of amenorrhea, fasting blood glucose (FBG) ranging between 100 mg/dL and 126 mg/dL, total cholesterol level ≥ 200 but less than 380 mg/dL, no consumption of medications known to influence carbohydrate and lipid metabolism, and no medical history involving hypo- or hyperthyroidism and liver or kidney disorders (Figure 1). Recruitment involved reaching out to potential candidates through Native American tribe health fairs and broader advertising initiatives. Before participation, participants were given detailed verbal and written explanations outlining the study and informed about the voluntary nature of their involvement and the confidential handling of their information. Upon consenting to participate in the study, participants underwent comprehensive medical assessments and detailed dietary history evaluations (Table 2).

### 2.3. Dietary Assessment and Anthropometric Measurement

Anthropometric assessments, including height, weight, and hip and waist circumferences, were conducted both at baseline and upon study completion (3 months). The waist-to-hip ratio was calculated using these measurements. Total body fat and lean mass content were determined using bioelectric impedance (Biodynamic Model 310e, Biodynamics Corp., Seattle, WA, USA), which evaluates the body’s impedance to electrical currents, correlating higher impedance with greater body fat content. Except for height, all other measurements were reassessed at monthly intervals throughout the study. The basal metabolic rate (BMR) was calculated by the Mifflin-St. Jeor equation [30].

The initial assessment of participants’ baseline daily nutrient intake utilized a validated food frequency questionnaire endorsed by the National Institutes of Health (NIH). Participants exhibiting weight gain during the study received counseling regarding their daily food intake and were advised to make simple adjustments to their caloric intake based on the collected food frequency questionnaire. Additionally, a 7-day physical activity questionnaire was administered to gather detailed information about participant activities.

To ensure compliance with the intervention, several measures were implemented: (1) participants were provided with monthly calendars to record their daily regimen intake; (2) unused dietary regimens were returned during monthly visits and new supplies were received along with a new calendar; and (3) random bi-monthly 24-h food recalls via phone were conducted to monitor adherence. At the end of three months of treatment, a blood draw, anthropometric measurements, physical activity evaluations, and dietary assessments were performed.

### 2.4. Blood Collection and Processing

Overnight fasting venous blood samples were collected in ethylendiaminetetraacetic acid (EDTA) and non-EDTA vacutainer tubes from each participant at the beginning and at the end of the study. All blood samples were placed on ice until processing. Subsequently, plasma and serum were separated by centrifuging the samples at 2500× *g* for 20 min at 4 °C, then aliquoted and stored at −80 °C until analyses. At the end of the study, all samples were analyzed simultaneously to minimize potential variability.

### 2.5. Plasma and Serum Analyses

Analysis of fasting blood glucose and glycated hemoglobin was performed using an ACE Clinical Analyzer (Monclair, NJ, USA). Prior to each test, calibration of the clinical analyzer was carried out using Gemcal reference serum (Alfa Wassermann, Inc.; West Caldwell, NJ, USA). Quality control was ensured by integrating Alfa Wassermann (QC-1) and (QC-2) in all tests to maintain accuracy. Fasting serum insulin levels were determined via the radioimmunoassay (RIA) method using a human insulin-specific RIA kit from LINCO Research (St. Charles, MO, USA). This specific RIA kit utilizes a double antibody technique to quantitatively measure insulin levels in blood or serum.

The serum samples were analyzed for total cholesterol, high-density lipoprotein (HDL), triglycerides, and lipoprotein-a levels using Alfa Wassermann kits (Alfa Wassermann, West Caldwell, NJ, USA). All lipid parameters, excluding low-density lipoprotein (LDL), were measured using the ACE Clinical Analyzer (Monclair, NJ, USA). The LDL was calculated by the Friedwald equation. Samples were also analyzed for hemoglobin by an automated combined impedance-light focusing hematology counter (Pentra Retic Hematology Instrument, ABX Diagnostics, Irvine, CA, USA).

### 2.6. Statistical Analyses

Treatment was the main plot factor, and baseline and final values were the repeated measures factors. Data were analyzed using SAS 9.4 (SAS Institute, Cary, NC, USA) using PROC GLIMMIX. Results are expressed as mean +/− SE. The significance of percentage differences between and within treatments was assessed using a paired Student’s *t*-test. The primary outcome variables were the change from baseline in fasting blood glucose, glycated hemoglobin, plasma insulin, and HOMA-IR. An analysis of variance (ANOVA)was used to assess treatment (flaxseed vs. flaxseed + fiber vs. control) differences. Significant differences were determined using the alpha level of 0.05.

## 3. Results

### 3.1. Subject Participation

Fifty-five Native American postmenopausal women were recruited to participate in the study. Out of the initial 55 participants recruited, 13 individuals discontinued their participation. A total of 9 women completed the control regimen, while 17 and 16 completed the flaxseed and flaxseed + fiber regimens, respectively. One participant withdrew from the flaxseed treatment group due to reported gastrointestinal discomfort. In response to complaints of gastrointestinal issues and food palatability concerns, guidance was provided to aid in integrating the study food into their diet, aiming to alleviate these problems. Among the reasons for dropout, four participants from the control group relocated. Eight subjects, four from the control regimen and two from each flaxseed treatment group, did not provide reasons for their withdrawal from the study (Figure 1). There were no differences in the baseline characteristics among the women who dropped out versus those who completed the study.

### 3.2. Nutrient Intake

Dietary analysis revealed no significant differences in baseline protein, carbohydrate, fiber, total fat, saturated fatty acids, polyunsaturated fatty acids, or calcium intake among the various groups, while there was a significant difference in caloric intake between the control and flaxseed groups (Table 3). Throughout the study duration, the subjects’ nutrient intake was monitored via 24-h phone-based food recalls (Table 4). Only total fat intake showed no notable changes across the study groups while they adhered to the study regimens. Comparing baseline to post-intervention for each study group (Table 5), it appeared that there was a significant increase in total energy for the control (*p* < 0.001) and flaxseed (*p* < 0.001) groups, carbohydrate for the control (*p* = 0.004) and flaxseed (*p* = 0.037) groups, dietary fiber for the flaxseed + fiber (*p* < 0.001) group, and calcium for the control (*p* =0.041) group. However, a clear decrease was recorded in polyunsaturated fatty acid intakes for the control (*p* < 0.001), flaxseed (*p* = 0.034), and flaxseed + fiber (*p* = 0.003) groups.

### 3.3. Anthropometric Measurements

The results of the anthropometric measurements are summarized in Table 6. The participants’ ages ranged from 47 to 62 years old. There were no significant changes in body weight after a 3-month adherence to the assigned dietary regimens. However, there was a tendency towards a decrease in body weight among subjects in the control group following the 3-month regimen (*p* = 0.07). Regarding the body mass index (BMI), no notable changes were observed among subjects in any of the treatment groups throughout the study duration. Interestingly, a significant difference in mean waist circumference was observed in the flaxseed group (*p* = 0.02), while no significant changes were noted in either the control or flaxseed + fiber groups. Additionally, the hip circumference notably decreased from baseline within the flaxseed group (*p* = 0.05), while no such change was observed in the other treatment groups. Furthermore, the waist-to-hip ratio exhibited a significant decrease from baseline in the control group (*p* = 0.01), contrasting with a significant increase noted in the flaxseed + fiber group (*p* = 0.01). Regarding the percentage of body fat, a significant increase was noted only among participants in the flaxseed + fiber treatment group (*p* = 0.01). The basal metabolic rate (BMR) showed a significant increase for the control group (*p* <0.001) and a significant decrease for the flaxseed (*p* = 0.002) and flaxseed + fiber (*p* < 0.001) groups.

### 3.4. Plasma and Serum Analyses

The glucose-related parameters are summarized in Table 7. Noticeably, consumption of 25–30 g of flaxseed did not yield significant effects on blood glucose, insulin levels, or HbA1c when comparing baseline values with their respective final values within the treatment groups. Conversely, a statistically significant increase in the final FBG values compared to baseline was observed in the control group (*p* = 0.01).

Analysis of final insulin values in comparison to baseline ones among the three groups did not reveal significant differences. Regarding glycated hemoglobin (HbA1c) values, no considerable changes were observed in the final values compared to the baseline across all the groups. Importantly, these values remained in the range of prediabetes limits (<6.5%) as defined by the World Health Organization. Additionally, the homeostatic model assessment of insulin resistance (HOMA-IR) values > 5 indicated insulin resistance in all three groups. Particularly, there was no discernible treatment effect observed in improving HOMA-IR among the subjects after 3-month regimens.

## 4. Discussion

The outcomes of the present study reveal that daily intake of approximately 30 g of whole ground flaxseed, when incorporated into bread, muffins, and energy drinks, did not yield significant changes in the final values of FBG, insulin, and HbA1c levels among Native American postmenopausal women. Conversely, the control group consuming white bread and oat muffins displayed a notable increase in FBG values, accompanied by an increase in insulin levels. These findings suggest that while the treatments involving flaxseed and flaxseed + fiber did not exert a measurable enhancement of the glycemic profile, they did demonstrate a noteworthy treatment effect. This effect was characterized by maintaining the glycemic profile relatively stable from baseline throughout the duration of the study. Given the absence of prior research investigating the impact of flaxseed on glucose status, specifically among Native American postmenopausal women, comparing the consistency of our study’s results with similar investigations remains challenging. Various studies have outlined differing thresholds for defining insulin resistance using HOMA-IR [31,32,33]. However, insulin resistance represents a complex condition influenced by various factors, including but not limited to overweight, hypertension, hypertriglyceridemia, hypo-HDL-cholesterolemia, and familial predisposition to diabetes and/or hypertension [31]. In our study, several factors, including overweight [31], postmenopausal status [34], and borderline hyperglycemia [31], were recognized as contributors to insulin resistance. The presence of such factors collectively contributed to the elevation of HOMA-IR values beyond the threshold of 2.77, indicating a state of insulin resistance among the study participants. Although impaired fasting glucose values were observed across all three groups, the HbA1c levels remained below 6.5%, indicating that all subjects maintained glycemic control. However, the discrepancy between lower glycemic index values and the observed impaired fasting glucose might have misled the interpretation of the results. It is imperative to note that utilizing glycated hemoglobin as a sole marker for assessing glycemic control has limitations, as highlighted in several studies [35,36,37]. The study by Matsumuto et al. revealed that very low values of HbA1c in diabetic patients are associated with genetic variants such as HbS, HbC, and HbD, which are prevalent in heterogeneous ethnic groups [31]. It is crucial to note that identifying the specific variant or derivative of Hb before or during testing could facilitate more precise HbA1c measurement by utilizing methods unaffected by the particular variant or derivative. Therefore, the aforementioned factors, including genetic variants, age-related influences, and technical challenges associated with measuring glycated hemoglobin in the presence of Hb variants, might have contributed to the observed lower HbA1c levels. This is noteworthy, especially considering that FBG levels were slightly elevated in all groups from baseline to the study conclusion. The observed sustaining effect of flaxseed on glucose homeostasis partially aligns with the outcomes of a previous study [38]. Indeed, they found that in postmenopausal women, daily intake of crushed flaxseed improved glucose and insulin levels as effectively as oral estrogen–progesterone therapy. It is notable that the administration method differed from our study, as Lemay et al. administered crushed flaxseed without incorporating it into bread and muffins [38]. This distinction in intervention methodologies suggests that the ingestion of crushed flaxseed alone, unlike our approach, might have significantly improved the glucose profile among postmenopausal women participating in their study. Similarly, the administration of raw milled flaxseed significantly reduced insulin resistance in the research conducted by Hutchins et al. [39], while consuming ground flaxseed and hesperidin improved glucose homeostasis and reduced insulin levels at the end of the study conducted by Yari et al. [40]. These studies highlight the potential benefits of flaxseed for glucose metabolism, though intake form and accompanying dietary factors may influence outcomes. Two other investigations found that flaxseed supplementation can significantly reduce FBG and improve HOMA-IR compared to wheat bran [41] or wheat flour [42] in bread form. However, the small sample sizes in both studies [43,44] limit the generalizability of these findings, highlighting the necessity for larger, more diverse studies to ascertain the robustness of these effects on a broader scale. The disparity in the form of intake, compared to our incorporation of flaxseed into muffins and bread, could potentially contribute to the observed differences. Furthermore, it is essential to consider that bread and muffins are recognized as high-glycemic index foods, potentially influencing the outcomes observed. There are other studies in the existing literature that corroborate our findings [43,44,45,46]. Notably, Javidi et al. reported that flaxseed supplementation did not yield significant reductions in fasting serum glucose, insulin concentrations, HOMA-IR, or beta-cell function [46]. The results of the investigation conducted by Juntunen et al. [43], which examined the consumption of wheat bread and lignin-rich rye bread [47], indicated no significant differences in plasma glucose and insulin levels between baseline and final measurements in either group. A decrease in plasma insulin values was noted during rye bread treatment (*p* = 0.993). These observations imply that the effects of flaxseed and flaxseed + fiber in our study were corroborating the sustaining impact seen with rye bread in maintaining the glycemic profile. However, a noteworthy observation in our study was a significant increase in the final fasting glucose values (*p* = 0.01) within the control regimen group, contrasting earlier findings from the other studies [43,44]. This discrepancy aligns with the literature suggesting that the inclusion of high-glycemic index foods [48,49] in the daily diet may contribute to elevated fasting blood glucose levels. Therefore, the consumption of wheat bread within our control group might have potentially contributed to the observed rise in fasting glucose levels. Particularly, there was a significant increase in caloric intake (*p* < 0.001) and carbohydrate intake (*p* = 0.004) in the control group when comparing baseline to post-intervention. These observations suggest that the increased intake of calories and carbohydrates may have influenced the fasting glucose levels [50,51]. However, while the type of bread appeared to impact fasting glucose levels within the control group, no notable alterations were observed in the fasting glucose, glycated hemoglobin, or insulin levels within the flaxseed and flaxseed + fiber groups. This leads to the suggestion that the incorporation of flaxseed [38,39,40,41,42] and fiber [52,53,54,55,56] might exhibit treatment effects in maintaining a stable glycemic profile. We acknowledge that the small sample size of the control group due to the high dropout rate might not allow solid and definitive conclusions to compare flaxseed and flaxseed + fiber treatments. Additionally, a significant difference in total energy intake was observed among the three groups at baseline. By the end of the study, all nutrient intakes were significantly different, except for total fat (*p* = 0.242). The disparities in the nutrient intakes can be attributed to the differences in the provided regimens, which varied in their amounts of carbohydrates, lipids, proteins, and other nutrients (Table 1). These variations likely contributed to the observed differences in outcomes. Regarding calcium intake, it appears that the only group showing a significant intake increase at the end of the study is the control group. Despite this notable rise, the expected positive impact on glucose metabolism, such as enhancing insulin sensitivity, was not observed. This lack of effect contradicts previous findings that suggest higher calcium intake can improve insulin sensitivity and support glucose homeostasis [57,58].

The basal metabolic rate (BMR) represents the energy expended by an organism at rest in order to maintain body functions, including metabolic homeostasis, breathing, heart rate, etc. [59]. Observing the variation in BMR from baseline to post-intervention, it appears that the control group experienced a significant increase in BMR, likely in response to the upward trend in energy and carbohydrate intake. Given that the study population consists of prediabetic individuals, this increase in BMR can be correlated with their level of glycemic imbalance, as evidenced by the elevated FBG at the end of the study. The mechanisms behind this increase include elevated carbohydrate oxidation, increased glucogenesis, and hepatic glucose output, as well as a reduced capacity for glycogen synthesis [59,60,61]. These metabolic responses are characteristic of prediabetic individuals, whose bodies may struggle to manage glucose efficiently, thus leading to increased energy expenditure to maintain metabolic homeostasis [60,61,62]. In contrast, the BMR significantly decreased for both flaxseed and flaxseed + fiber groups. This reduction could be attributed to the metabolic benefits of flaxseed and fiber. The high fiber content in these diets may also promote satiety, leading to reduced overall caloric intake and, consequently, a lower BMR [60].

The same two regimens—flaxseed and flaxseed + fiber—were associated with the highest intakes of total fat and saturated fatty acids, with a significant increase in saturated fatty acids by the end of the study. Emerging evidence indicates that increased consumption of total fat and saturated fatty acids worsens carbohydrate metabolism by elevating the production of free fatty acids. These fatty acids subsequently compete with glucose uptake by muscles. The excess availability of plasma free fatty acids, derived from dietary sources, accumulates not only in adipose tissue but also in non-adipose tissues like the liver and muscles, leading to a decrease in intracellular glucose concentration, resulting in hyperglycemia. To counterbalance this condition, pancreatic beta cells respond by producing excessive insulin, leading to hyperinsulinemia. Prolonged hyperinsulinemia can negatively affect beta cell function, ultimately contributing to insulin resistance [63,64,65,66]. Regarding the PUFA intake at the end of the study, it is evident that the study regimens provided high amounts of PUFAs, as reported in their nutrient composition in Table 1. Notably, the flaxseed + fiber group exhibited the highest PUFA intake (Table 4). The baseline compared to the post-intervention PUFA intake highlighted that the observed decrease is significant for all study groups (Table 5). The recent literature has emphasized that the quality of dietary fat plays a pivotal role in influencing insulin sensitivity and increased insulin resistance [67,68,69,70,71], whereas polyunsaturated fatty acids (PUFAs) have been associated with enhanced insulin sensitivity [72,73,74,75]. Noticeably, it has been documented that saturated fatty acids constitute a significant dietary factor in the progression of type II diabetes among specific ethnic populations, including Native Americans [76,77], Japanese Americans [78], and Mexican Americans [79]. In our study, the high intake of total fat and saturated fatty acids might have overshadowed the beneficial effects of flaxseed and flaxseed + fiber observed in the treatment groups. This was further compounded by a significant decrease in the overall intake of PUFAs among participants. The treatment that incorporated flaxseed into participants’ daily diet showed a significant decrease in total cholesterol and LDL cholesterol levels by approximately 7% and 10%, respectively. These improvements in lipid profiles are likely due to the high content and quality of PUFAs in flaxseed, along with its other beneficial components.

The glycemic index of plant-based foods, particularly grains, is notably lower when they are less refined. It is commonly believed that incorporating whole kernels into baked goods like bread and muffins can enhance their glycemic and insulin profiles, especially when compared to higher fiber content alone [44]. Processing methods such as heating, cooking, baking, and the size or structure of grains [43,48,80] may significantly influence their glycemic index [44]. Consequently, the baking process might elevate the glycemic index of breads, impacting both postprandial and fasting glucose levels. However, baking bread at lower temperatures for extended periods might yield better outcomes in glucose regulation by impeding bread digestion and promoting the formation of resistant starch and the retrogradation of amylase [44]. Hence, in our study, processed flaxseed incorporated into breads and muffins was theoretically expected to be more effective in reducing glucose levels and improving insulin responses compared to ground flaxseed alone. Despite these theoretical expectations, findings from an earlier study by Leinonen et al. using whole kernel rye bread suggest that the intact structure of the kernel may rupture during the baking process, exposing it to enzymatic processes, leading to a lack of improvement in the glycemic profile observed in the treatment groups [44]. As previously discussed, the physical form of food significantly impacts the glycemic response [81]. It has been demonstrated that the intake of pasta (white spaghetti) notably reduced blood glucose levels compared to white and whole-meal bread [49]. Similar findings were documented in another study, where white and whole-meal spaghetti notably decreased glycemic responses in contrast to white bread and semolina bread [82]. These observations emphasize the influence of various non-nutrient factors on the glycemic index of food, consequently affecting the overall glycemic profile.

Our study might have yielded more robust outcomes had we integrated ground flaxseed with low-glycemic index foods like bread and muffins. Therefore, it is plausible to speculate that the inclusion of flaxseed might mitigate hyperglycemia and hyperinsulinemia when incorporated into other foods with lower glycemic indexes, as opposed to bread and muffins, which generally register higher on the glycemic index scale. The findings from our study indicate that the consumption of 25 to 30 g of flaxseed incorporated into baked products by postmenopausal Native American women over a 3-month period is associated with the maintenance of their glycemic profile. These findings highlight the complex interaction between food processing methods and glycemic responses. While whole grains and certain preparation methods theoretically enhance glycemic and insulin profiles, their practical applications, such as baking, can alter these benefits. The influence of non-nutrient factors, such as the physical form of food and cooking methods, must be carefully considered when designing dietary interventions aimed at improving glucose metabolism. Understanding these nuances can help optimize dietary recommendations and improve metabolic health outcomes.

### Limitations of the Study

This study has several limitations that should be noted. The primary limitation is the lack of specific hemoglobin variants or derivatives during recruitment and measurement, which would have allowed for more precise adjustments and accurate HbA1c readings. Additionally, the form of flaxseed intake (incorporated into high-glycemic index foods like bread and muffins) may have confounded the impact of flaxseed on glucose status in postmenopausal Native American women. The high glycemic index of these foods, combined with the baking process, could have mitigated the positive effects of flaxseed, making it difficult to discern its true benefits. Furthermore, the high dropout rate and the small number of participants in the control group compared to the treatment groups undermine the statistical power of the analysis, limiting the generalizability of the findings. These limitations suggest the need for more specific studies focusing on the impact of flaxseed intake on postmenopausal Native American women, using alternative forms of flaxseed intake that do not involve high-glycemic index foods, and incorporating detailed identification of hemoglobin variants to enable more accurate assessments of glucose control.

## 5. Conclusions

This investigation sought to assess the potential effects of daily flaxseed incorporation into the diet over a 3-month period on glucose regulation in postmenopausal Native American women. The outcomes revealed no statistically significant improvements in fasting blood glucose, insulin, or glycated hemoglobin (HbA1c) values. Despite the absence of statistical significance, the treatments involving flaxseed and flaxseed + fiber exhibited a discernible trend towards exerting a stabilizing effect on glucose control within the studied population. The implications of our findings suggest that the inclusion of flaxseed, especially when integrated into low-glycemic foods, may have a mitigating effect on hyperglycemia and hyperinsulinemia. Furthermore, investigating the potential impact of raw flaxseed intake spread throughout the day could reveal more pronounced effects on glycemic control and glucose homeostasis. Acknowledging inherent limitations, such as genetic identification constraints and variations in intake forms, our study emphasizes the imperative for further in-depth investigations into the nuanced effects of flaxseed. Noteworthy is the observed elevation in carbohydrates intake for the control group, total energy intake for the control and flaxseed groups, and saturated fatty acid intake in the flaxseed and flaxseed + fiber treatments, potentially confounding the true impact of flaxseed on various glucose status parameters. Nevertheless, it remains crucial to conduct complementary studies that explore the impact of flaxseed intake on glycemic control across diverse participant subsets, particularly considering ethnic groups and their genetic particularities. These research endeavors are integral to fostering a more comprehensive understanding of the nuanced effects of flaxseed on glucose regulation and facilitating the tailoring of dietary recommendations for specific populations.

## Figures and Tables

**Table 1:** Amount of flaxseed incorporated and nutrient composition of study supplements.

Treatment	Bread			Muffin			Powder
	Control	Flaxseed	Flaxseed + Fiber	Control	Flaxseed	Flaxseed + Fiber	Flaxseed
Flaxseed amount incorporated (g)	-	1.5	1.5	-	5	5	8
Serving size	1 slice (=38 g)			1 piece (=70 g)			1 Tbsp (=8 g)
Energy (kcal)	70	80	70	150	160	130	55
Carbohydrates (g)	12	16	7	25	22	21	9
Protein (g)	2	3	8	5	4	4	5
Lipids (g)	1	1	4	4.5	6	4.5	3
n-3 PUFA (g)	>0.05	>0.3	>0.3	>0.04	>1.02	>1.01	>3.3
n-6 PUFA (g)	>0.02	>0.7	>0.7	>0.1	>2.62	>2.26	>8.3
Fiber (g)	0.5	3	4	1	2	5	6

Tbsp: tablespoon; PUFA: polyunsaturated fatty acid; n-3 PUFA: omega-3 polyunsaturated fatty acid; n-6 PUFA: omega-6 polyunsaturated fatty acid. Values are as mentioned on the product labels provided by Natural Oven Bakery, Manitowic, WI, USA.

**Table 2:** Baseline clinical parameters of the included participants.

	Control	Flaxseed	Flaxseed + Fiber
Glucose (mg/dL)	101.4 ± 3.8	104.3 ± 2.6	101.0 ± 4.5
Total cholesterol (mg/dL)	216.1 ± 11.6	232.1 ± 9.8	232.1 ± 10.5
Hemoglobin (g/dL)	14.4 ± 0.4	14.3 ± 0.8	15.1 ± 0.7

Data represent the least squares mean ± SE.

**Table 3:** Baseline nutrient intake assessed by a food frequency questionnaire.

Measures	Control	Flaxseed	Flaxseed + Fiber	*p* Value
Total energy (kcal)	1123 ± 104 ^a^	1683 ± 300 ^b^	1784 ± 684 ^ab^	0.036
Nutrients (g)				
Protein	46.6 ± 14.3	64.7 ± 47.2	58.6 ± 25.1	0.455
Carbohydrates	136.4 ± 41.6	194.7 ± 106.3	229.5 ± 91.4	0.059
Dietary fiber	12.7 ± 2.4	18.5 ± 13.4	16.4 ± 6.1	0.423
Total fat	44.6 ± 16.8	77.3 ± 67.5	74.3 ± 31.2	0.242
Saturated fatty acids	11.5 ± 3.4	22.6 ± 17.4	22.6 ± 11.2	0.059
Polyunsaturated fatty acids	10.1 ± 2.3	17.2 ± 16.3	17.4 ± 8.2	0.277
Minerals (mg)				
Calcium	366.4 ± 129.3	661.2 ± 417.1	647.8 ± 449.4	0.157

Data represent the least squares mean ± SE. Values in the same line with different subscript letters are significantly different at *p* < 0.05.

**Table 4:** Average nutrient intake during the study using 24-h food recalls.

Measures	Control	Flaxseed	Flaxseed + Fiber	*p* Value
Total energy (kcal)	1511 ± 193 ^a^	1994 ± 161 ^b^	1967 ± 161 ^b^	<0.001
Nutrients (g)				
Protein	46.2 ± 8.4 ^a^	66.8 ± 5.4 ^b^	69.0 ± 6.3 ^b^	<0.001
Carbohydrates	195.4 ± 31.9 ^a^	252.4 ± 25.9 ^b^	218.3 ± 19.4 ^a^	<0.001
Dietary fiber	12.5 ± 5.3 ^a^	21.8 ± 8.3 ^b^	30.4 ± 10.0 ^c^	<0.001
Total fat	49.0 ± 27.3	77.9 ± 24.4	71.1 ± 28.0	0.430
Saturated fatty acids	11.9 ± 3.0 ^a^	20.3 ± 2.1 ^b^	20.4 ± 3.3 ^b^	<0.001
Polyunsaturated fatty acids	5.0 ± 2.1 ^a^	8.4 ± 1.3 ^b^	10.5 ± 2.2 ^c^	<0.001
Minerals (mg)				
Calcium	475.8 ± 72.1 ^a^	814.3 ± 67.6 ^b^	654.1 ± 66.2 ^c^	<0.001

Data represent the least squares mean ± SE. Values in the same line with different subscript letters are significantly different at *p* < 0.05.

**Table 5:** The *p* values of the ANOVA statistical comparison of caloric and nutrient intake changes between the baseline and post-intervention levels.

	Control	Flaxseed	Flaxseed + Fiber
Total energy (kcal)	<0.001	<0.001	0.306
Protein	0.943	0.306	0.118
Carbohydrates	0.004	0.037	0.635
Dietary fiber	0.919	0.394	<0.001
Total fat	0.686	0.973	0.762
Saturated fatty acids	0.795	0.592	0.457
Polyunsaturated fatty acids	<0.001	0.034	0.003
Calcium	0.041	0.145	0.956

**Table 6:** Age, anthropometric measurements, and the BMR throughout the study with flaxseed supplementation in Native American postmenopausal women.

	Control			Flaxseed			Flaxseed + Fiber		
	Baseline	Final	*p* Value	Baseline	Final	*p* Value	Baseline	Final	*p* Value
n	17	9	-	20	17	-	18	16	
Age, yrs	50.8 ± 3.1	-	-	57.0 ± 2.2	-	-	60.4 ± 2.5	-	-
Weight, kg	75.58 ± 5.72	72.83 ± 5.76	0.071	82.34 ± 4.04	81.82 ± 4.06	0.591	79.99 ± 4.29	79.35 ± 4.29	0.510
BMI, kg/m^2^	29.00 ± 1.96	28.12 ± 1.99	0.139	31.11 ± 1.39	31.11 ± 1.40	0.998	31.17 ± 1.47	30.96 ± 1.47	0.551
BMR (kcal)	1350.17 ± 6.14	1483.17 ± 5.83	<0.001	1555.27 ± 4.62	1550.07 ± 4.18	0.002	1498.50 ± 4.13	1492.10 ± 4.56	<0.001
Hip circumference, inches	42.43 ± 1.73	43.28 ± 1.73	0.304	44.71 ± 1.14	43.45 ± 1.18	0.051	43.90 ± 1.17	43.08 ± 1.20	0.146
Waist circumference, inches	36.39 ± 1.93	36.03 ± 1.93	0.608	39.25 ± 1.23	37.93 ± 1.26	0.020	39.86 ± 1.30	39.62 ± 1.31	0.580
Waist/hip ratio	0.84 ^a^ ± 0.02	0.82 ^b^ ± 0.02	0.013	0.86 ± 0.01	0.86 ± 0.01	0.414	0.90 ^a^ ± 0.01	0.92 ^b^ ± 0.01	0.011
Body fat, %	37.99 ± 1.94	35.91 ± 2.10	0.268	39.52 ± 1.37	38.60 ± 1.45	0.483	40.45 ^a^ ± 1.48	43.75 ^b^ ± 1.48	0.011
Fat body weight	29.35 ± 3.48	26.83 ± 3.56	0.134	33.36 ± 2.46	32.55 ± 2.50	0.471	33.02 ± 2.62	34.87 ±2.62	0.123
Lean body weight	46.23 ± 2.85	46.40 ± 2.88	0.857	49.34 ± 2.01	49.52 ± 2.03	0.772	45.81 ± 2.14	45.10 ± 2.14	0.290

BMR: basal metabolic rate calculated by the Mifflin-St. Jeor equation; BMI: body mass index. Values in the same line for the same treatment (Control, Flaxseed, and Flaxseed + Fiber) with different subscript letters are significantly different at *p* < 0.05.

**Table 7:** Effects of a three-month flaxseed supplementation on serum glucose parameters in Native American postmenopausal women.

Control					Flaxseed				Flaxseed + Fiber			
	Baseline	Final	*p* Value	% Change from Baseline	Baseline	Final	*p* Value	% Change from Baseline	Baseline	Final	*p* Value	% Change from Baseline
Glucose (mg/dL)	91.4 ± 4.90 ^a^	102 ± 5.10 ^b^	0.014	12.47	104.3 ± 2.60	104 ± 2.90	0.300	0	101 ± 4.50	106 ± 4.90	0.200	5
Insulin (µU/mL)	19.38 ± 5.30	31.44 ± 5.60	0.120	62.22	14.4 ± 4.20	17.62 ± 4.20	0.590	22.36	16.97 ± 4.60	17.14 ± 4.80	0.900	1
P_A_1c_	5.80 ± 0.16	5.80 ± 0.10	0.911	0	5.7 ± 0.1	5.9 ± 0.1	0.466	3.5	5.6 ± 0.14	5.7 ± 0.14	0.770	1.78
HOMA-IR	5.80 ± 0.34	6.55 ± 0.35	0.130	12.93	5.79 ± 0.26	5.87 ± 0.26	0.840	1.38	5.64 ± 0.32	5.86 ± 0.38	0.660	3.9

Data represent the least squares mean ± SE. Differences were considered significant at *p* < 0.05. n = 9 for the control group, n = 17 for the flaxseed group, and n = 16 for the flaxseed with additional fiber group. P_A_1c_ = percentage of hemoglobin; HOMA-IR = homeostatic model assessment of insulin resistance. Values in the same line for the same treatment (Control, Flaxseed, and Flaxseed + Fiber) with different subscript letters are significantly different at *p* < 0.05.

## Data Availability

Data is contained within the article.
